# Zoledronate and Clodronate affecting bone repair in non-critical defects grafted with carbonated nanostructured hydroxyapatite: an *in vivo* study fostering clinical reasoning

**DOI:** 10.2340/biid.v12.45131

**Published:** 2025-12-17

**Authors:** Carlos Henrique Sardenberg Pereira, Gustavo Vicentis Oliveira Fernandes, Adriana Terezinha Neves Novellino Alves, Alexandre Malta Rossi, José Mauro Granjeiro, Mônica Diuana Calasans-Maia, Gutemberg Gomes Alves

**Affiliations:** a Dentistry School, Fluminense Federal University, Niterói, Brazil; b Missouri School of Dentistry and Oral Health, A.T. Still University, St. Louis, MO, USA; c Department of Condensed Matter, Applied Physics and Nanoscience, Centro Brasileiro de Pesquisas Físicas (CBPF), Urca, Brazil; d Bioengineering Sector, INMETRO, Xerém, Duque de Caxias, Brazil; e Oral Surgery Department, Fluminense Federal University, Niterói, Brazil; f Molecular and Cell Biology Department, Fluminense Federal University, Niterói, Brazil

**Keywords:** Bisphosphonates, clodronate, zolendronate, carbonated hydroxyapatite, femur, *in vivo*study

## Abstract

**Objective:**

This study aimed to compare the effect of two different generations of bisphosphonates (BPs) on bone repair assisted by an alloplastic bone graft (spheres of nanostructured carbonate apatite/calcium, CHA) in a non-critical defect in the rat femur.

**Materials and Methods:**

Thirty-six female Wistar rats were randomly assigned into six groups: Control group (blood clot), carbonate apatite (CHA) alone, Zoledronate (Zol) with blood clot, Clodronate (Clo) with blood clot, Zol+CHA, and Clo+CHA. Drugs were administered intraperitoneally (Zol: 0.6 mg/kg; Clo: 20 mg/kg) every 30 days for 60 days before surgery. Standardized monocortical femoral defects (2 mm) were created and filled according to group assignment. After 30 days of healing, samples were harvested for histological and histomorphometrical evaluation. New bone formation and remnant biomaterial were quantified. Statistical analysis included the Kruskal–Wallis test and Dunn’s post hoc (*p* < 0.05), along with Pearson and Spearman correlation analyses.

**Results:**

Histological analysis revealed enhanced new bone formation in groups treated with BPs, especially when combined with CHA. The Zol+CHA group exhibited the highest new bone formation (24.0 ± 4.0%), significantly greater than Control (2.0 ± 0.5%; *p* = 0.011) and CHA (5.0 ± 1.2%; *p* = 0.0017). The Clo and Clo+CHA groups also showed significant improvements (19%) compared to the Control (*p* = 0.03) and CHA (*p* = 0.04). Remnant biomaterial was significantly greater in Zol+CHA (15.0 ± 2.0) and Clo+CHA (15.0 ± 2.0%) than in CHA alone (8.0 ± 1.0%; *p* = 0.022), suggesting inhibition of bone graft resorption by BPs. Correlation analysis revealed a strong positive association between remnant biomaterial and new bone formation (Spearman ρ = 0.94, *p* = 0.005; Pearson *r* = 0.88, *p* = 0.021), supporting the biological synergy of CHA and BPs in bone repair.

**Conclusion:**

Both bisphosphonates enhanced bone repair in the femoral defect model, demonstrating a synergistic effect when combined with nanostructured CHA. Zoledronate required the presence of the biomaterial to exert its osteogenic influence, while Clodronate stimulated new bone formation independently. These findings indicate that generation-specific differences among bisphosphonates may guide their future use in bone tissue engineering strategies.

## Introduction

Bisphosphonates (BPs) are a well-established class of antiresorptive drugs used to prevent and treat various bone-related disorders, including Paget’s disease, hypercalcemia, osteolytic lesions associated with multiple myeloma, pathological fractures, osteoporosis, osteopenia, and bone metastases from malignancies [1–5]. BPs act primarily by suppressing osteoclastic activity, thereby reducing bone turnover and fracture risk. BPs are generally classified into non-nitrogenous (first-generation, e.g., Clodronate) and nitrogen-containing (second- and third-generation, e.g., alendronate, pamidronate, zoledronate) compounds, which differ in potency and mechanism of action [6, 7]. Non-nitrogenous BPs induce osteoclast apoptosis through toxic adenosine triphosphate (ATP) analogues, while nitrogenous agents inhibit the mevalonate pathway, disrupting osteoclast function and survival [1, 2, 4, 8].

Beyond their systemic antiresorptive properties, BPs have shown potential to modulate local bone repair [9]. Experimental and clinical studies suggest that controlled local delivery of BPs may enhance bone graft stability and reduce resorption, supporting their possible use as adjuncts in regenerative therapies [10–16]. However, excessive systemic exposure – particularly to potent nitrogen-containing BPs like zoledronate – has been associated with medication-related osteonecrosis of the jaw (MRONJ), underscoring the need for cautious translation of preclinical findings [6, 9, 17–20].

Among bone substitutes, hydroxyapatite (HA) is widely recognized for its osteoconductivity but is limited by slow resorption and incomplete remodeling [21]. In contrast, carbonate-substituted hydroxyapatite (CHA) more closely mimics the mineral composition of native bone, exhibiting improved solubility, bioactivity, and turnover potential [22–27]. Nanostructured CHA formulations further enhance these properties by increasing surface area and biological reactivity [28–31].

While prior studies have examined BP-enhanced grafts, the comparative effects of different BP generations when combined with a resorbable CHA scaffold remain unclear. To address this gap, this study aimed to evaluate the impact of BPs from two different generations, Zolendronate (Zol, 3^rd^ generation) and Clodronate (Clo, 1^st^ generation), on bone repair in non-critical femoral defects in rats filled with nanostructured CHA. The positive hypothesis was that combining BPs with CHA would improve bone repair in a generation-dependent manner, with distinct effects on bone formation and biomaterial resorption.

## Materials and methods

All procedures were approved by the Ethics Committee for Animal Use at the University (*anonymous file*) (Protocol n.436) and conducted in accordance with institutional and international guidelines for animal research (ARRIVE guidelines). A total of 36 healthy female Wistar rats (*Rattus norvegicus*), aged approximately 120 days and weighing an average of 250 g, were included in the study. The animals were housed in ventilated plastic cages (six per cage) under controlled conditions (22°C, standard light/dark cycle), with free access to a standard rodent diet (Nuvilab, Brazil) and filtered water *ad libitum*. Animals were randomly assigned to groups using a computer-generated sequence and coded containers to prevent allocation bias.

### Sample size and experimental design

Sample size (*n* = 6 per group) was based on prior pilot data detecting ≥30% differences in bone formation with 80% power and α = 0.05. Then, animals were randomly assigned to six experimental groups (*n* = 6 per group): G1. Group Control, bone defect filled with a blood clot only; G2. Group C**HA**, defect filled with carbonate apatite (CHA) biomaterial; G3. Zol, animals treated intraperitoneally with Zoledronate (0.6 mg/kg, every 30 days), defect filled with blood clot; G4. Zol+CHA, animals treated intraperitoneally with Zoledronate (0.6 mg/kg, every 30 days), but defect filled with CHA; **G5. Clo**, animals treated intraperitoneally with Clodronate (20 mg/kg, every 30 days), defect filled with blood clot; G6. Clo+CHA: animals treated intraperitoneally with Clodronate (20 mg/kg, every 30 days), but defect filled with CHA.

### Biomaterial and surgical procedure

Carbonate apatite (CHA) microspheres (400 μm < ⌀ < 500 μm), synthesized via low-temperature precipitation (5°C), with a low-crystallinity nanostructure, a Ca/P molar ratio of 1.67, and particle size <20 nm, were used in this experimental study. Surgical interventions were performed after 60 days of drug administration; Zoledronate (Novartis Pharma AG, Basel, Switzerland) was applied intraperitoneally (0.6 mg/kg, every 30 days – a total of three applications), and the Clodronate (Jenahexal Pharma GmbH, Thuringia, Germany) was applied intraperitoneally (20 mg/kg, every 30 days – total of three application). Both were diluted in sterile saline immediately prior to injection [32].

General anesthesia was induced with intramuscular ketamine (75 mg/kg) and xylazine (1.5 ml/kg). A longitudinal incision was made over the right femur, and a 2 mm diameter monocortical bone defect was created using a spherical carbide bur (Jet AIR 2) with a handpiece at 1200 RPM, under constant irrigation. Defects were filled according to group allocation using a standardized curette (Lucas n.12), and soft tissues were closed with 5-0 nylon sutures (Ethicon^®^, Johnson & Johnson, U.S.A.).

### Euthanasia and sample collection

After 30 days of bone healing, animals were euthanized by intraperitoneal overdose of ketamine (75 mg/kg) and xylazine (5 mg/kg). The right femur was harvested and fixed in 10% buffered formalin. Samples were decalcified, embedded in paraffin, sectioned at 5 μm, and stained with hematoxylin and eosin (HE).

### Histological and morphometrical analysis

Histological evaluation was performed on six randomly selected fields per sample at 40× and 400× magnification using a Zeiss Axio Imager Z1 microscope (Zeiss, Göttingen, Germany). Scoring followed the criteria for assessing the presence of pre-existing bone, newly formed bone, medullary tissue, biomaterial, and other tissue components (e.g. adipose tissue, vasculature). Quantitative histomorphometry involved calculating the percentage of new bone (NB) area and the remnant biomaterial area. Histological and histomorphometric analyses were performed by a calibrated examiner blinded to group allocation.

Areas of pre-existing bone, newly formed bone, medullary tissue, and biomaterial were segmented using color thresholding in ImageJ (v1.54), applying standard hue-saturation-brightness ranges validated in previous studies for hematoxylin-eosin (HE)-stained bone tissue.

### Statistical analysis

Statistical analysis was conducted using GraphPad InStat 5.0 and Prism 8.0 (GraphPad Software, U.S.A.). Non-parametric data were analyzed using the Kruskal–Wallis test followed by Dunn’s post hoc test. A significance level of *p* < 0.05 was considered statistically significant. Results are presented as mean ± standard deviation (SD).

To assess the relationship between the amount of remnant biomaterial and the extent of newly formed bone, correlation analyses were conducted using both Pearson’s correlation coefficient (to evaluate linear relationships) and Spearman’s rank correlation coefficient (to assess monotonic relationships, less sensitive to outliers and distributional assumptions). These tests were applied to two datasets: (1) a subset of groups treated with biomaterial (CHA, Zol+CHA, Clo+CHA) and (2) the entire sample of six experimental groups, including those without biomaterial (Control, Zol, Clo). Correlation coefficients and *p*-values were calculated to determine the strength and significance of the associations, with *p*-values < 0.05 considered statistically significant.

## Results

All animals remained healthy throughout the experiment, with no evidence of infection or surgical complications. Macroscopically, healing proceeded uneventfully across all groups.

### Descriptive histology

**Figure 1:**
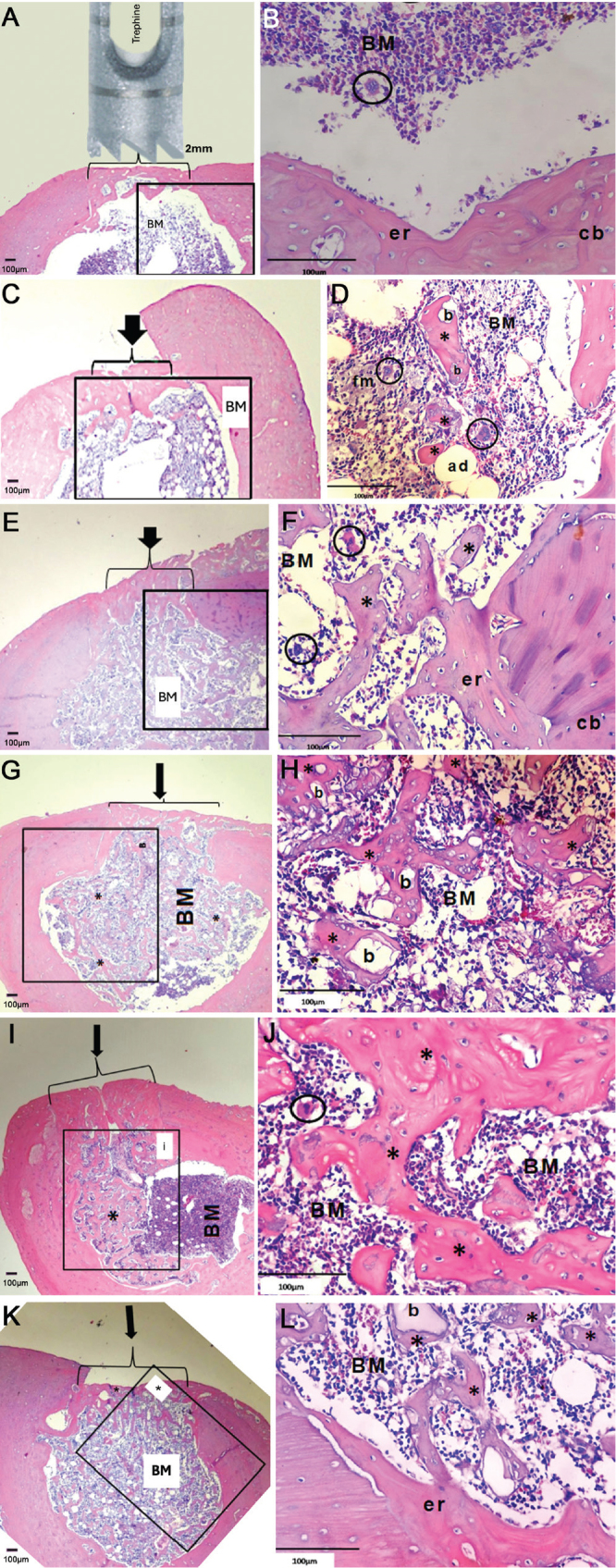
Representative histological images of femoral defects from different experimental groups. **(A, B) Control**: Defect completely bridged by compact bone (cb) enclosing marrow (BM) with endosteal reaction (er) and megakaryocytes (circles). **(C, D) CHA**: Bone bridge formation with intramedullary bone (*) surrounding biomaterial (b); marrow containing adipocytes (ad), foamy macrophages (fm), and megakaryocytes. **(E, F) Zol**: Defect filled with newly formed trabeculae (*) and compact bone (cb); marrow with megakaryocytes and endosteal reaction. **(G, H) Zol+CHA**: Bone bridge with abundant trabecular bone (*) partially encompassing biomaterial (b). **(I, J) Clo**: Defect filled with mature bone; marrow interspersed with trabeculae (*) and interface between native and endosteal bone (i). **(K, L) Clo+CHA**: Mature bone formation with delicate trabeculae (*) associated with biomaterial (b) and endosteal reaction (er). (*Stained with hematoxylin-eosin; magnifications: 40× for A, C, E, G, I, K and 400× for B, D, F, H, J, L*).

Thirty days after grafting the biomaterial, histological evaluations showed that the defect area in the Control group was filled by a thin bony bridge, indicating that the defect was not critical. Additionally, a large amount of medullary tissue, surrounded by compact bone, was observed. In all experimental groups, inflammatory infiltrates were barely seen, with little endosteal reaction and the presence of megakaryocytes (Figure 1A, B).

The CHA group (Figure 1C, D) presented a defect area filled by a thicker bone bridge compared to the Control group (Figure 1A, B). It can also be observed that considerable amounts of bone marrow tissue comprise bone marrow cells, as well as the presence of adipocytes, foamy macrophages, and megakaryocytes. The medullary cavity lies surrounded by compact bone, with areas of bone formation toward the intramedullary direction, and islands of newly formed bone surrounding the biomaterial (Figure 1C, D). CHA was used in the form of small particles, indicating that the spheres of biomaterial have been fragmented and/or partially resorbed.

In the Zol group, it was also observed that the area of the defect was filled with a bone bridge, along with a small amount of medullary tissue comprising bone marrow cells. It can be observed that medullary tissue was found intermingled with large amounts of dispersed newly formed bone trabeculae, with the presence of a small strip of endosteal new bone formation (Figure 1E, F). When animals treated with Zolendronate received the grafted biomaterial (Zol + CHA group), the same pattern was observed, with a small amount of bone marrow tissue interspersed with large amounts of dispersed newly formed bone trabeculae (Figure 1G, H), similar to the Zol group. However, in this group, it became evident that this newly formed bone appeared to encompass the biomaterial (Figure 1G, H) and was more evenly distributed inside the medullary cavity. The biomaterial appeared as particulate fragments, even though larger than in the CHA group.

In the Clo group, the defect area was filled with mature bone. Newly formed bone trabeculae were interspersed present in almost half of the medullary cavity, surrounded by compact bone, with a small strip of endosteal new bone formation observed (Figure 1I, J). In the group treated with biomaterial (Clo + CHA), a very similar pattern to that seen in the Clo group was observed, with a defect area filled with mature bone. Still, in the form of delicate trabeculae of newly formed bone, throughout the area of the medullary cavity, in a strong association with the biomaterial. Here, as well as in the Zol group, CHA is in the form of apparently larger particles than those found in CHA. The presence of endosteal reaction (Figure 1K, L) was observed.

### Histomorphometric analysis

**Figure 2:**
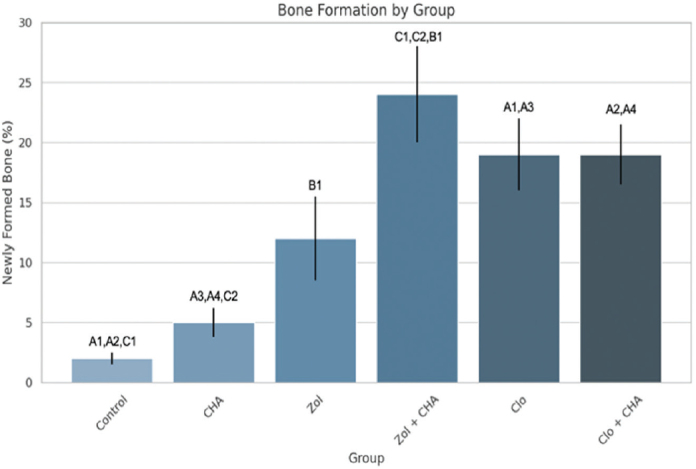
Histomorphometrical analysis of the newly formed bone. (A = *p* < 0.05; B = *p* < 0.01; C = *p* < 0.001).

The Control group showed 2% (±0.5%) of bone formation, followed by 5.0% (±1.2%) in the CHA group. The Zol group experienced a sixfold increase in mean bone formation compared to the Control group (Cohen’s *d* = 4.7, ns), although this difference was not statistically significant. The combination Zol+CHA produced the highest new bone formation (24.0 ± 4.0%), with a very large effect versus Control (Cohen’s *d* = 9.5, *p* = 0.0011) and CHA (Cohen’s *d* = 7.0, *p* < 0.0017). Clo and Clo+CHA groups both showed about ninefold higher bone formation than Control (Cohen’s d ≈ 7.5–8.1, *p* = 0.03) (Tables 1 and 2; Figure 2), confirming a strong treatment effect. It can be concluded for new bone formation that the Zol + CHA group demonstrated the highest statistically significant results; both Clo and Clo+CHA also significantly outperformed Control and CHA, but not Zol or Zol+CHA, and, interestingly, Zol alone was not significantly better than Control, likely due to variability (SD = 3.5) and small sample size (*n* = 6).

**Table 1:** Newly formed bone (% area) and the statistical correlation.

Group	Mean ± SD (%)	Versus control	Versus CHA	Versus Zol	Versus Zol+CHA	Versus Clo	Versus Clo+CHA
Control	2.0 ± 0.5	—					
CHA	5.0 ± 1.2	ns	—				
Zol	12.0 ± 3.5	ns	ns	—			
Zol+CHA	24.0 ± 4.0	*p* = 0.0011	*p* < 0.0017	*p* = 0.013	—	ns	ns
Clo	19.0 ± 3.0	*p* = 0.037	*p* = 0.046	ns	ns	—	ns
Clo+CHA	19.0 ± 2.5	*p* = 0.033	*p* = 0.042	ns	ns	ns	—

ns: non-significant result; SD: standard deviation.

**Table 2:** Approximate effect sizes (Cohen’s *d*) and magnitude interpretations for new bone.

Comparison	Mean difference (%)	P-value	Pooled SD	Cohen’s *d*	Interpretation
Zol+CHA vs. Control	22.0	0.0011	≈2.3	9.5	Very large
Zol+CHA vs. CHA	19.0	<0.0017	≈2.7	7.0	Very large
Clo vs. Control	17.0	0.037	≈2.3	7.4	Very large
Clo+CHA vs. Control	17.0	0.033	≈2.1	8.1	Very large
Zol vs. Control	10.0	ns	≈2.1	4.7	Large (but not significant)

ns: non-significant result; SD: standard deviation.

**Figure 3:**
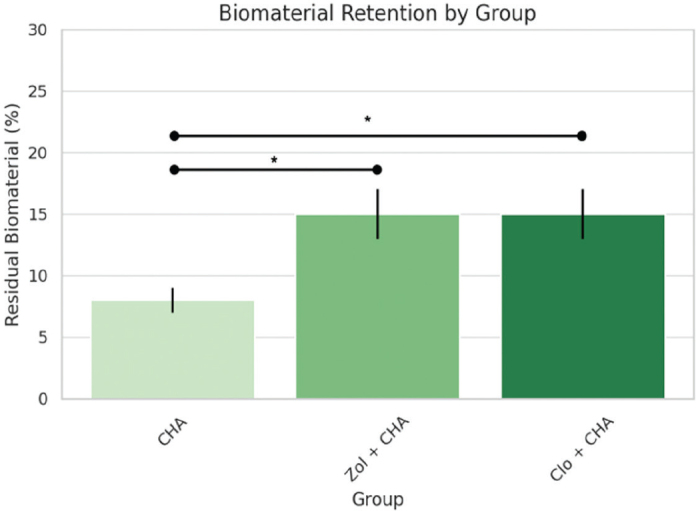
Histomorphometrical analysis of the percentage of biomaterial found after the trial period. (* = *p* < 0.05).

For remnant biomaterial, Zol+CHA and Clo+CHA groups retained nearly double the CHA area (15%) compared to CHA alone (8%), with large effect sizes (Cohen’s *d* = 4.4, *p* = 0.022 for both). This suggests BP treatment substantially reduced material resorption. Thus, this increase presented a statistically significant difference when compared to the CHA group (*p* = 0.022), but no statistically significant difference was found between the drug-treated groups (Tables 3 and 4, Figure 3). It is possible to conclude that treatment with BPs (Zol or Clo) combined with CHA led to significantly greater biomaterial remnant compared to CHA alone.

**Table 3:** Remnant biomaterial (% area) and the statistical correlation.

Group	Mean ± SD (%)	Versus CHA	Versus Zol+CHA	Versus Clo+CHA
CHA	8.0 ± 1.0	—		
Zol + CHA	15.0 ± 2.0	*p* = 0.022	—	ns
Clo + CHA	15.0 ± 2.0	*p* = 0.022	ns	—

ns: non-significant result; SD: standard deviation.

**Table 4:** Approximate effect sizes (Cohen’s *d*) and magnitude interpretations for remnant biomaterial.

Comparison	Mean difference (%)	P-value	Pooled SD	Cohen’s *d*	Interpretation
Zol+CHA vs. CHA	7.0	0.022	≈1.6	4.4	Very large
Clo+CHA vs. CHA	7.0	0.022	≈1.6	4.4	Very large

**Figure 4:**
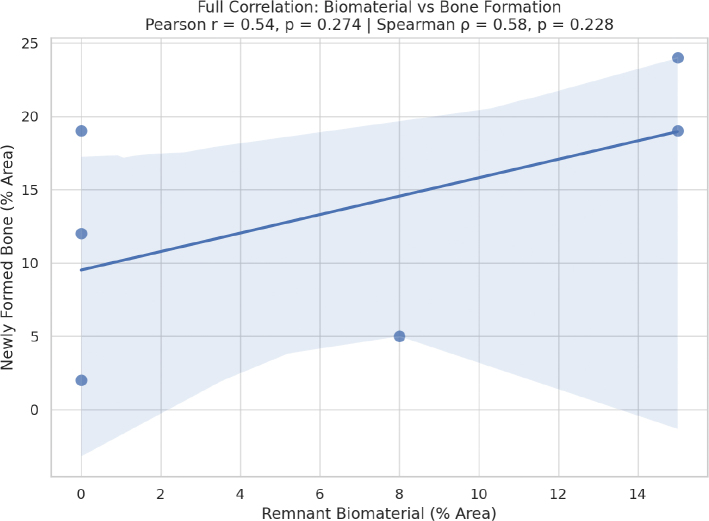
Correlation chart for all evaluated groups.

For the correlation analysis across all groups, Pearson correlation was *r* = 0.88 (*p* = 0.021) and Spearman correlation was ρ = 0.94 (*p* = 0.005). These results confirm a strong and statistically significant correlation between the amount of remnant biomaterial and the extent of new bone formation, even when considering all six groups (Figure 4).

**Figure 5:**
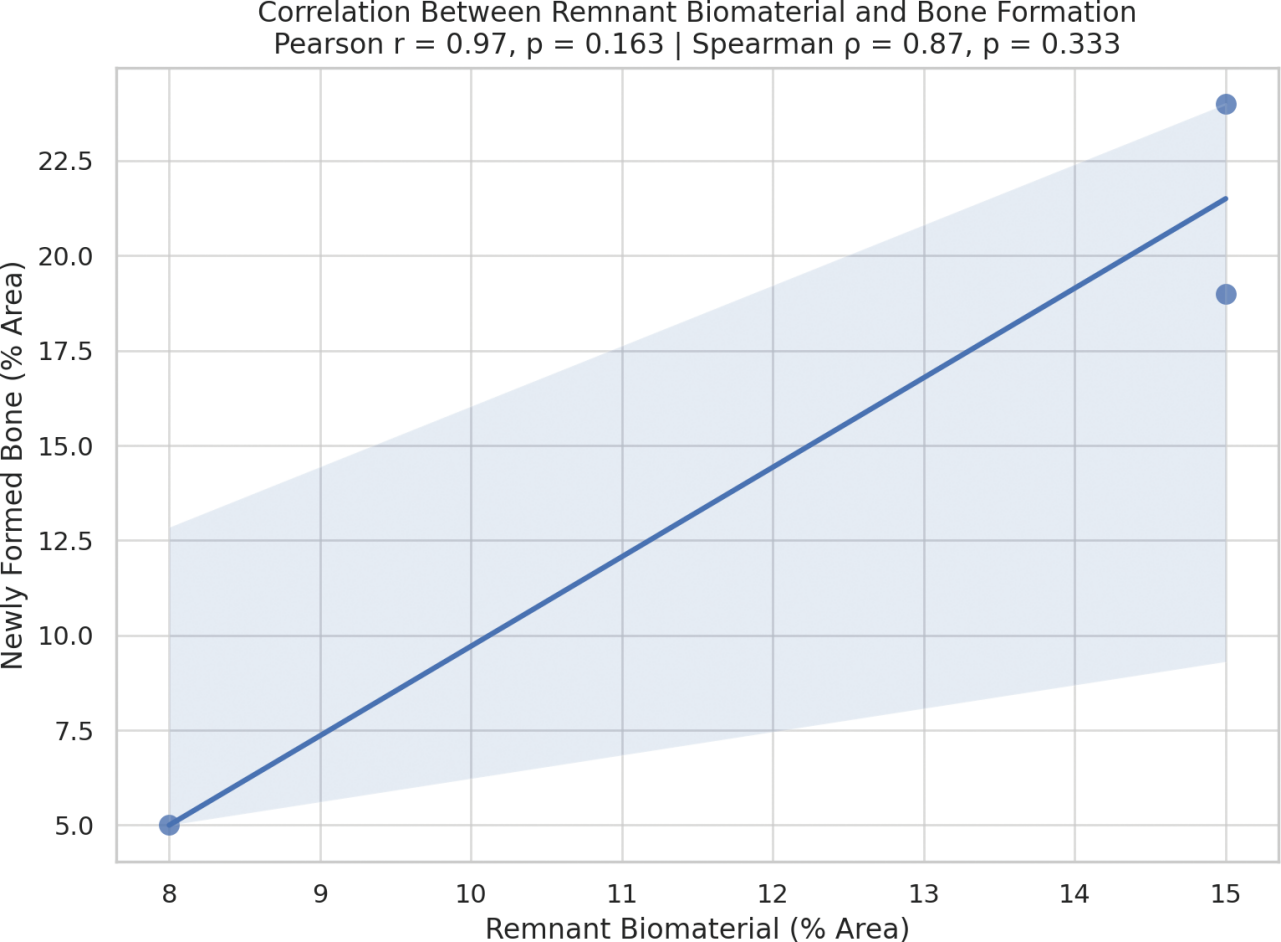
Correlation graphic between remnant biomaterial and newly formed bone for the biomaterial-treated groups.

Evaluating the specific correlation between remnant biomaterials and new bone, Pearson and Spearman correlations were higher, respectively, *r* = 0.96 (*p* = 0.090) and ρ = 1.00 (*p* = 0.0001). These results confirm a strong positive correlation between the amount of biomaterial retained and the extent of new bone formation, especially in terms of monotonic (Spearman), indicating that greater biomaterial remnant is associated with increased bone repair (Figure 5). The slightly higher Spearman coefficient suggests a strong monotonic relationship, which is not strictly linear, reflecting the biological variability typical of bone healing responses.

## Discussion

This study compared the effects of two BPs from different generations on bone formation when combined with a CHA biomaterial scaffold. The results demonstrated that CHA associated with BPs enhanced bone formation compared with CHA alone or untreated controls in a non-critical bone defect model in rats. These findings align with previous studies showing that BPs, when combined with bone grafts, can improve bone formation and reduce graft resorption, suggesting a synergistic relationship between these agents and biomaterials in bone repair contexts [33, 34]. However, these results should be viewed as preclinical evidence rather than proof of clinical efficacy, as this experimental model does not address long-term remodeling, biomechanical performance, or systemic effects.

Research has shown that the interaction between BPs and biomaterials can vary widely. While some studies report no major changes in osteogenesis following localized or systemic BP administration [3, 35, 36], others demonstrate enhanced bone formation and reduced graft resorption [12–14]. In this study, Zoledronate (a third-generation BP) produced a marked increase in bone formation, both quantitatively and qualitatively, when combined with CHA. In contrast, Clodronate (a first-generation BP) showed a more modest effect – stimulating bone formation independently but without significant quantitative gains when paired with CHA. Nonetheless, qualitative analyses indicated that new bone in the Clodronate group appeared more dispersed and closely associated with CHA particles.

Previous studies corroborate these observations: Zoledronate has been shown to promote bone formation when administered with biomaterials or autogenous grafts in animal models [37–41]. These findings support the concept that BPs can serve as useful adjuncts to enhance biomaterial-based bone repair [39]. Moreover, the development of ‘smart’ biomaterials – such as CHA scaffolds functionalized with BPs – offers promising directions for tissue engineering and controlled drug delivery [42].

Caution, however, is warranted due to the risk of medication-related osteonecrosis of the jaw (MRONJ), particularly when nitrogen-containing BPs are used at high systemic doses [1–5, 43, 44]. In this study, Zoledronate and Clodronate were applied locally at low concentrations, well below those associated with MRONJ in animal or human models [18]. No histological evidence of osteonecrosis or delayed healing was observed, supporting the safety of this experimental approach. While Zoledronate displayed higher osteogenic potential, Clodronate’s safer pharmacological profile – without documented osteonecrotic effects, even at elevated doses – makes it an attractive option for further exploration with bioresorbable scaffolds [34].

Indeed, incorporating Clodronate into biomimetic calcium phosphate materials has been shown to create an environment favorable for bone formation [45, 46]. In this study, although Clodronate did not quantitatively enhance CHA-mediated bone formation, it maintained osteoconductive behavior, with new bone closely surrounding the biomaterial. This suggests that Clodronate-CHA combinations may offer synergistic benefits warranting further study.

Another important observation was that both BPs studied appeared to influence CHA resorption. In untreated groups, CHA fragments were smaller, suggesting that the drugs slowed biomaterial degradation [47, 48]. This effect likely stems from the antiresorptive action of BPs on osteoclasts, modulating the RANK (Receptor Activator of Nuclear factor Kappa-B)/RANKL (Receptor Activator of Nuclear factor Kappa-B Ligand)/OPG (Osteoprotegerin) signaling pathway [49]. Future studies incorporating immunohistochemical and molecular analyses could clarify how these mechanisms contribute to the observed outcomes.

Although this study did not directly investigate molecular pathways, it is plausible that BPs modulated osteoclast activity and subsequent osteoblast signaling through the RANKL/OPG axis or integrin-mediated adhesion, thereby enhancing mineralized tissue deposition. Future investigations should verify these hypotheses using gene expression profiling or immunohistochemistry, enabling a deeper understanding of how first- and third-generation BPs differentially influence bone repair.

### Clinical perspective

The present findings indicate that both Zoledronate and Clodronate can enhance bone repair when combined with CHA scaffolds. However, as these results are derived from controlled animal experiments, they must be interpreted as hypothesis-generating rather than clinically definitive. Translation to human applications will require careful consideration of pharmacokinetics, bone metabolism, and interspecies differences.

The observed synergy between Zoledronate and CHA suggests potential clinical utility in bone repair procedures such as extraction sockets, peri-implant defects, and atrophic ridge augmentation. Given CHA’s chemical similarity to bone and its moderate resorption rate, its combination with locally delivered BPs could preserve bone volume and accelerate healing, particularly in patients with impaired bone metabolism (e.g. osteoporosis or cancer-related bone loss). Nevertheless, clinical application must balance enhanced bone formation with the potential risk of MRONJ. Zoledronate offers greater osteogenic potency but a higher risk profile, whereas Clodronate may provide a safer, albeit milder, alternative.

If validated in dose-response and long-term safety studies, BP-functionalized CHA scaffolds could become valuable tools for patients with reduced regenerative capacity – such as postmenopausal women, individuals under antiresorptive therapy, or those requiring minor bone augmentation.

### Experimental model: Bone defect choice

A 2 mm monocortical femoral defect was chosen as a standardized, non-critical-size model to study early biological behavior of CHA under BP influence. This design minimized fracture risk and ensured consistent defect creation across animals, enabling reliable histological and morphometric comparisons. Critical-size defects in rats (5–8 mm) carry a higher mechanical risk and require osteoinductive intervention to heal [50–56]. In contrast, non-critical defects (2–4 mm) can spontaneously repair, making them suitable for evaluating early healing and biocompatibility under physiological conditions [21, 57].

As emphasized by Schemitsch [58], the concept of ‘critical size’ is functional rather than absolute – it depends on species, bone geometry, and vascularization. Thus, smaller, site-specific models such as the one used here remain valid for studying localized biological responses to biomaterials and drugs, even if they are not ‘critical’ in the traditional sense.

### Limitations of the study

This study has several limitations. The use of healthy Wistar rats with small, non-critical defects may not fully represent clinical bone repair conditions, particularly in patients with systemic diseases or large osseous defects. The sample size (*n* = 6 per group) limited statistical power, and evaluation was restricted to a single 30-day time point, precluding conclusions about long-term outcomes or late adverse effects such as osteonecrosis. Additionally, only histological and morphometric analyses were performed; molecular and immunohistochemical evaluations, particularly of osteoclast activity, angiogenesis, and signaling pathways, were not conducted. Finally, the absence of a comparative critical-size defect group limits the assessment of CHA and BP performance under more demanding tissue repair conditions.

Future research should employ larger animal models, multiple time points, and combined molecular analyses to validate the translational potential of CHA-BP composites. Including both critical and non-critical defect models will help define the true tissue repair thresholds for these bioactive materials.

## Conclusion

In this study, it is possible to conclude that both bisphosphonates enhanced new bone formation, with Zoledronate effective only in combination with CHA, and with Clodronate demonstrating osteogenic potential independently. These findings are preliminary and warrant validation in larger animal models and long-term human studies before clinical translation.

## Key messages

Both Zoledronate and Clodronate significantly improved bone formation compared to controls

Clodronate alone achieved approximately 19% new bone formation, nearly matching the performance of Clodronate with CHA

The association of bisphosphonates and biomaterials had significantly higher remnant biomaterial than CHA alone.

## Data Availability

All data were included in the article.
